# The Boy Captive of the Texas-Mier Expedition

**Published:** 1912-04

**Authors:** 


### Books and Magazines.

The Boy Captive of the Texas-Mier Expedition.—By Fannie Chambers Gooch-Iglehart. Author of "Face to Face With the Mexicans;" "Christmas in Old Mexico," and other short stories, sketches and essays. Revised, reprinted and republished by the author. Illustrations by Bock. In two styles of binding. By mail, $1.25 and 75c. Address Ursuline Convent, San Antonio, Texas.

This remarkable book has received the highest commendation from leading educators throughout the United States and from the press, even in London. It is regarded as a valuable contribution to the history of Texas, heretofore unpublished, and has been adopted in many of the high schools and graded schools as a supplementary text-book on Texas history. Aside from its historical value, it is a thrilling and intensely interesting narrative, and holds the reader entranced from beginning to end; one can scarcely put it down. The tale—actual occurrences—is told by the accomplished author in a fascinating style, and it is replete with humor, yet moves almost to tears, with pathos and human sympathy. The Texas Medical Journal recommends it to parents of boys as a story that will incite to high ideals of courtesy, courage, fidelity and personal honor. All boys should read it; it sets a model in ethics and morals worthy of emulation. The reading of it will make of the boys better, stronger, purer, abler men, and hence improve the race. It is a preliminary, therefore, in the science of eugenics. F. E. D.

